# Comparison of Virtual Non-Contrast Images Generated by Spectral Detector Computed Tomography and Conventional Computed Tomography Images of Histologically Confirmed Hepatic Pathologies in 28 Dogs

**DOI:** 10.3390/ani15233366

**Published:** 2025-11-21

**Authors:** Lydia K. Claußen, Alkje M. van Gemmeren, Philipp Lietz, Sebastian Meller, Adriano Wang-Leandro, Andreas Beineke, Verena Nerschbach, Holger A. Volk, Kristina Merhof

**Affiliations:** 1 Department of Small Animal Medicine and Surgery, University of Veterinary Medicine Hannover, 30559 Hannover, Germany; 2 Department of Pathology, University of Veterinary Medicine Hannover, 30559 Hannover, Germany

**Keywords:** spectral detector computed tomography (SDCT), virtual non-contrast (VNC), dual-energy, spectral-based images, true unenhanced (TUE) images, hepatopathy

## Abstract

Spectral detector computed tomography (SDCT) is an innovative imaging technique in veterinary medicine that utilises simultaneous data acquisition at different energy levels using two rows of detectors. This technique provides several interesting applications which improve insights into tissue composition. One implementation is the generation of virtual non-contrast (VNC) images from post-contrast spectral CT data by identifying and subtracting iodine pixels. Preliminary studies suggest that VNC images may offer diagnostic quality comparable to true unenhanced (TUE) images in healthy dogs; however, this technique has yet to be evaluated in clinical patients. This study compared the Hounsfield units (HUs) measured in VNC to those in TUE images of canine hepatic pathologies, taking into account specific types of pathologies based on their imaging characteristics. The attenuation values of the VNC and TUE series were analysed using two one-sided *t*-tests (TOST), and the signal-to-noise ratio (SNR) was calculated for each region of interest (ROI). A 5-point Likert scale was utilised to assess image noise, quality, and iodine subtraction in the VNC images. A total of 287 ROIs were analysed in the liver, gallbladder, paravertebral muscle, and pancreatic body of 28 dogs with histopathologically confirmed hepatic pathologies. 92.61% of the hepatic ROIs displayed a “negligible” difference of ≤10 HUs between VNC and TUE images, with significant *p*-values of <0.05 maintained for all ROIs within the limit of ≤10 HUs in the TOST, confirming equivalence between the two imaging modalities. The image quality assessment indicated that SDCT-derived images provided equal or superior quality compared to conventional CT. Therefore, it can be concluded that VNC images calculated from SDCT data could be an alternative to conventional TUE images for hepatic pathologies.

## Simple Summary

Liver diseases are common in dogs and often require advanced imaging techniques for diagnosis and treatment. In traditional computed tomography, a pre- and post-contrast scan is routinely performed. With detector-based spectral computed tomography, virtual non-contrast (VNC) images generated from post-contrast spectral data can eliminate the need for scanning the patient twice. This method could reduce examination time, radiation exposure, and the duration animals spend under general anaesthesia if it proves to be clinically viable. In this study, we examined the equivalence of Hounsfield units calculated by the VNC algorithm compared to those from the true unenhanced (TUE) series, as well as the quality of the VNC images in dogs with liver disease. Our results showed that the differences in attenuation values between the two techniques were minor and did not impact diagnostic assessment. In fact, image quality was equal to or better than that of the conventional series. These findings suggest that virtual non-contrast images could potentially serve as an alternative to TUE images for hepatic pathologies in canine patients, while still providing accurate diagnostic information.

## 1. Introduction

The computed tomography (CT) features of canine liver lesions have been thoroughly documented in veterinary literature [1,2,3,4,5,6], with comparisons to other imaging methods also reported [7,8,9]. However, the gold standard for classifying liver lesions remains liver biopsies, as Burti et al. highlighted a significant misclassification rate of 38% when distinguishing between benign and malignant hepatic lesions using CT [5]. Therefore, CT is currently regarded as an adjunct to liver biopsies for characterising hepatic pathologies.

The potential benefits of tissue characterisation through acquiring CT data at different energy levels were recognised as early as the 1970s [10,11], when two scans with different X-ray energy spectra had to be performed. However, clinical use has been limited by increased radiation exposure and technical challenges such as longer acquisition times, which make images more susceptible to motion artefacts [12] and higher noise levels in low-kilovoltage images [13]. Recent advancements in CT technology have addressed these issues, particularly through the simultaneous detection of photons at different energy levels within a single scan. In the detector-based spectral CT technique, two distinct detector layers record photons of different energies: the superficial layer detects lower-energy photons, while the deeper layer records higher-energy photons [14,15]. This approach has revitalised interest in spectral detector computed tomography (SDCT) in recent years. By exploiting the different ways photons interact with matter at various energies, SDCT can collect dual-energy data, yielding improved insights into tissue composition and enhanced characterisation of pathological lesions [16]. In human medicine, promising studies have demonstrated the improved tissue characterisation of SDCT for liver pathologies [17,18,19,20].

SDCT also enables the creation of virtual non-contrast (VNC) image sets from post-contrast scans by identifying and subtracting the iodine pixels, which could potentially eliminate the need for routine true unenhanced (TUE) CT scans in the future [21]. In human medicine, numerous studies have shown that VNC images are comparable to TUE images, providing similar quality and information for both physiological [15,22,23,24,25] and pathological tissues. It is already recommended to omit the native pre-contrast scan for certain regions [26,27,28,29,30,31,32,33,34].

Progenitor studies at our institution examined the equivalence of VNC to TUE series derived from SDCT in healthy dogs [35] and in cases of splenic pathologies [36]. The results were similar to those seen in human medicine, showing negligible differences between VNC and TUE images in the quantitative analysis of healthy organs and a high reliability of the VNC algorithm for structurally altered spleens. Our study aimed to determine whether similar findings could also be observed in various liver pathologies. If VNC images are proven reliable across different organs and conditions, pre-contrast scans might be unnecessary in future protocols. This could considerably reduce radiation exposure, scan duration, and the need for general anaesthesia in veterinary patients [35]. Another study conducted at our institution already explored the use of the VNC technique in rabbits, which benefit from shorter scan times due to their sensitivity to general anaesthesia, sedation, and stress when scanned awake [37].

The VNC algorithm exhibited a potential weakness in subtracting contrast medium from small vessels [22,24], as well as in some mineralised lesions [36,38] and pathologies including fat-attenuating tissues [15,24,25,35]. From a technical perspective, VNC images should offer better image quality compared to conventional images due to improved spatial and temporal resolution, reduced noise, and reduced artefacts. As mentioned earlier in our previous study, streaking artefacts caused by incomplete mixing of contrast medium persisted in VNC images and could compromise image quality [36].

The present study has two objectives: First, to determine the equivalence of the HUs calculated by the VNC algorithm compared to those measured in TUE images in structurally altered livers of canine patients. Second, to ascertain whether certain types of lesions, based on their imaging characteristics, as well as specific pathologies, influence the precision of attenuation values in VNC images and to evaluate if this technique could be applied in a clinical setting. We hypothesise that attenuation values in VNC images are accurate for hepatic pathologies in general, as well as for different types of lesions based on imaging features and for various pathologies based on histopathology. Furthermore, we aim to assess the image quality of the studies quantitatively by the evaluation of the signal-to-noise ratio (SNR) as a measure of the meaningful signal to the background noise and qualitatively and hypothesise that the quality of SDCT images is superior to that of conventional CT images.

## 2. Materials and Methods

### 2.1. Study Design and Population

This retrospective study was carried out in the Department of Small Animal Medicine and Surgery at the University of Veterinary Medicine Hannover. Medical records of patients were reviewed from July 2021 to July 2023 for dogs diagnosed with structural changes in the liver detected on CT images. Dogs were included if they underwent an SDCT of the abdomen, and the liver lesion was characterised through histopathological examination. Signalment, including age, breed, sex, body weight, and the histopathological diagnosis of liver lesions, was documented. Since the data used were obtained during routine clinical practice, and the owner consented to the use of clinical data for future research, no additional ethical committee approval was required for this study.

### 2.2. CT Examinations

All dogs were given intravenously induced general anaesthesia and were subsequently positioned in head-first sternal recumbency on the patient table. Throughout the CT scans, anaesthesia was maintained using isoflurane CP^®^ (CP-Pharma Handels-Gesellschaft mbH, Burgdorf, Germany) inhalation. The CT examinations were carried out with a Spectral-Detector CT Scanner (IQon Spectral CT, Philips Healthcare Germany, Hamburg, Germany), which has up to 256 slices and a rotation speed of 0.27 s, following these scan parameters: a maximum tube potential of 120 kV, automatic mAs depending on the standard protocol for different patient weight categories (range 174–320 mAs), a pitch of 0.6, a gantry rotation speed of 0.5 s, a slice thickness of 2 mm, and a matrix of 512 images. CT scans followed standardised protocols for the abdomen, utilising soft tissue and bone kernels with the appropriate bone window (window level: 800; window width: 2000) and soft tissue window (window level: 60; window width: 350). After pre-contrast scanning, Xenetix^®^ 350 (Guerbet GmbH, Sulzbach, Germany) contrast medium was administered intravenously into the cephalic vein via a Power-Dualinjector-System (MEDRAD Stellant, Bayer HealthCare, Leverkusen, Germany) at a dose of 2 mL/kg (700 mg Iobitridol/kg) with an administration time of 30 s. The administration speed depended on the contrast agent volume required for each patient. Image acquisition of the portal venous phase started with a 60-s delay after positive feedback from the bolus tracking software with the ROI set in the thoracic aorta, and a trigger HU of 150, or, if bolus tracking failed, the scan was initiated manually 60–70 s after contrast media administration.

### 2.3. Imaging Features of Liver Lesions

A Diplomate of the European College of Veterinary Diagnostic Imaging (KM) who was blinded to the clinical, surgical, and histopathological findings, evaluated all available CT studies in a soft tissue window using commercially available DICOM imaging viewing software (OsiriX^®^ MD v 9.0.1, Pixmeo SARL, Bernex, Switzerland). The examiner was allowed to adjust the window width and level to enhance lesion visibility. Multiplanar reconstruction was used for measurements in three planes. The images were assessed for the presence and degree of peritoneal effusion, as well as the surface and contours of the liver. Hepatic alterations were categorised as diffuse, focal, or multifocal. The following imaging characteristics were documented for focal and multifocal lesions: number of lesions, localisation, shape, borders, margination, extent, size of the largest lesion in all three planes, capsule formation, cavitation, attenuation compared to surrounding hepatic parenchyma before and after contrast medium administration, enhancement pattern, and degree of contrast enhancement compared to paraspinal muscle on the same transverse image level. The paraspinal musculature was used to assess the degree of contrast enhancement of liver lesions, as a comparison to the surrounding unaffected liver parenchyma was not possible in diffusely altered organs. The presence of mineralisation was evaluated for all lesions, including diffuse hepatopathies. Additionally, the portal lymph nodes were assessed for size, structure, enhancement pattern, and degree of enhancement (Table 1).

### 2.4. Quantitative Image Analysis

The quantitative image analysis was performed by a board-certified radiologist (KM), a veterinary radiology trainee (PL), and a clinical veterinarian (LC) using a software approved for medical image analysis (IntelliSpace Portal Version 11.x/Philips Healthcare Germany, Hamburg, Germany) on a monitor also certified for image analysis. TUE images were linked and synchronised with three reconstructions derived from post-contrast data, including conventional post-contrast, VNC, and monoenergetic images at 70 keV. The monoenergetic images at 70 keV were used, as this specific energy level increases the visibility of contrast agents significantly due to improved contrast and reduced noise [39,40,41]. 70 keV corresponds to an image equivalent to the conventional 120 kVp [42].

The examiners were allowed to adjust the window width and level to enhance lesion visibility. Blinding was unnecessary as the different reconstructions and the TUE images could be easily distinguished. Multiple circular regions of interest (ROIs) were placed in the TUE images and then transferred to the post-contrast series using the copy-and-paste function to ensure consistent size and localisation. Where feasible, a uniform area of 1 cm^2^ ± 0.05 cm^2^ was utilised; for structures with smaller dimensions, the largest possible circular ROI was employed. ROIs were positioned as follows: paravertebral muscle at the level of the 13th thoracic vertebra (1 ROI), pancreatic body (1 ROI), gallbladder content (1 ROI), and liver (6–12 ROIs, Table 2).

The paraspinal muscle and pancreas showed the best results regarding the accuracy of Hounsfield units in VNC images compared to TUE images in our progenitor study [35]. The gall bladder was additionally selected because its content should not have contrast uptake, and there should be no significant difference in Hounsfield units between VNC and TUE images. Larger differences between Hounsfield units of VNC and TUE images for muscle and pancreas compared to the previous study, and larger differences regarding the gall bladder content, could indicate a technical error.

**Figure 1:**
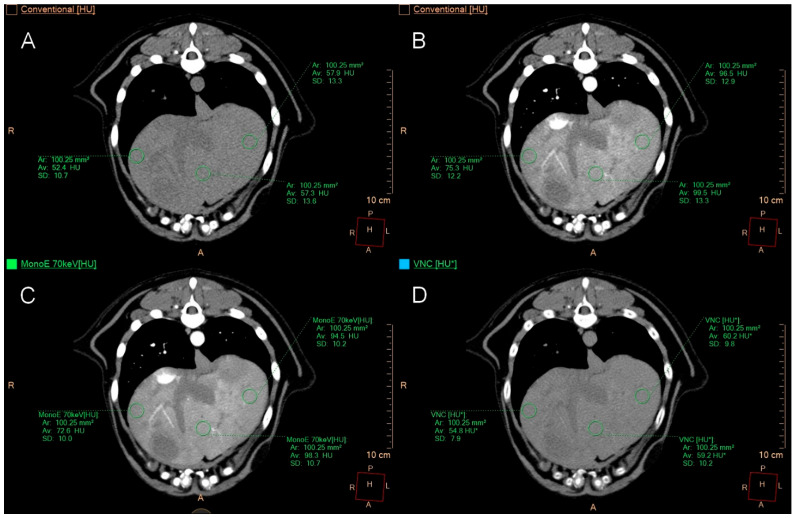
Transverse CT images of a liver with diffuse changes. Image (**A**) shows conventional images, image (**B**) presents conventional images post-contrast, image (**C**) features monoenergetic images at 70 keV post-contrast, and image (**D**) displays VNC images. ROIs were placed in the left and right medial liver lobes and one centrally; the remaining ROIs were positioned further caudally. The patient exhibited lymphohistiocytic inflammation and vacuolar degeneration on histopathology.

In cases of a uniform appearance of the hepatic parenchyma, one ROI was positioned in the left lateral lobe, left medial lobe, right lateral lobe, right medial lobe, and two in the central region, avoiding larger vessels (Figure 1).

**Figure 2:**
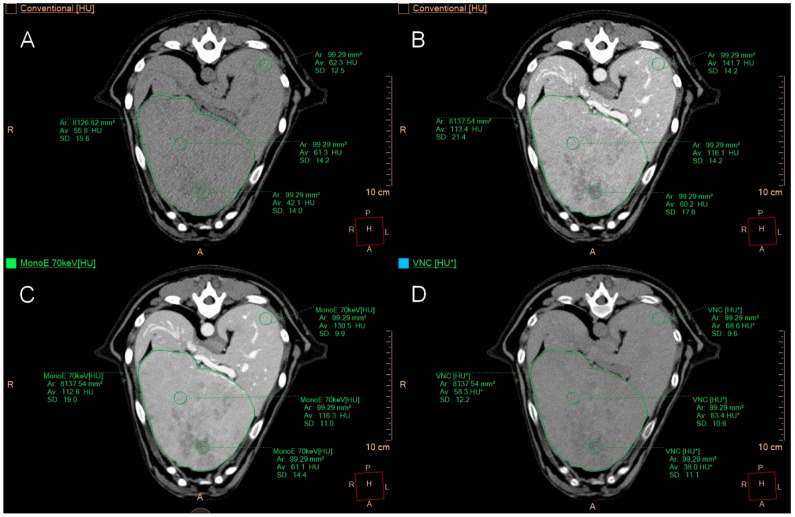
Transverse CT images of a liver with a focal lesion. Image (**A**) displays conventional images, image (**B**) shows post-contrast images, image (**C**) presents monoenergetic images at 70 keV post-contrast, and image (**D**) features VNC images. A polygonal ROI was placed at the largest transverse cross-section of the lesion. Additionally, two central ROIs are positioned in areas with different attenuations. The ROI in the left dorsal aspect of the liver represents one of the ROIs drawn in the more normal-appearing hepatic parenchyma. The patient was found to have hepatocellular carcinoma on histopathology.

If the organ had only one focal lesion, a polygonal ROI was positioned at the widest transverse diameter of the lesion, with two ROIs located in the periphery, two in the centre, and two in the normal-appearing parenchyma at the maximum possible distance from the lesion. For a single heterogeneous lesion, one of the centrally positioned ROIs was placed in the vascularized area and the other ROI in the cavernous or less well-perfused area (Figure 2).

**Figure 3:**
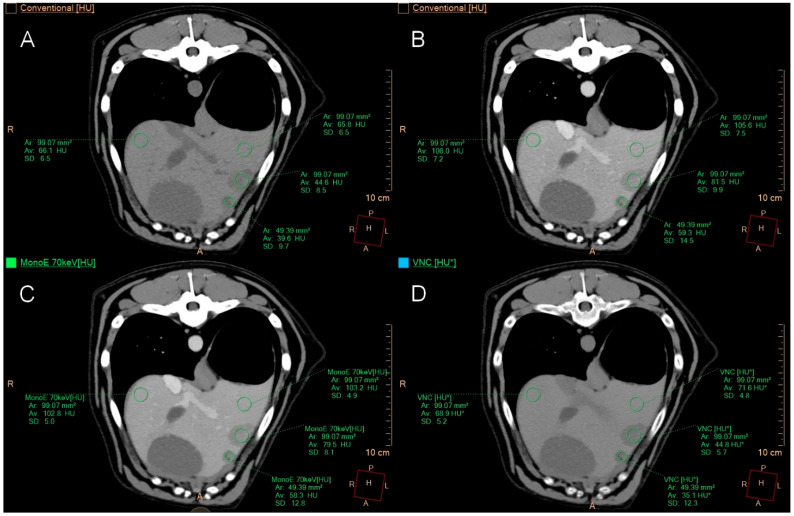
Transverse CT images of a liver with multifocal pathology. Image (**A**) shows conventional images; image (**B**) displays conventional images post-contrast; image (**C**) presents monoenergetic images at 70 keV post-contrast; and image (**D**) features VNC images. Two regions of interest (ROIs) are placed in two of the multifocal lesions (ventrolateral location of the left medial liver lobe), with one ROI measuring 1 cm^2^ and the other having the largest possible diameter. The remaining two ROIs are positioned in normal-appearing hepatic parenchyma (dorsally in the left and right medial liver lobes). The patient had cholangiocellular carcinoma confirmed by histopathology.

In cases of multifocal hepatopathy, the same measurements were taken for the largest lesion. When the multifocal lesions were very small, one ROI was placed per lesion (four in total), and two additional ROIs were positioned in the surrounding hepatic parenchyma (Figure 3).

Mean attenuation values in Hounsfield units (HUs) and the standard deviation (SD) were recorded for each ROI. The signal-to-noise ratio (SNR) was determined by dividing the HUs by the respective SD. The difference in HU values was calculated for each paired ROI in VNC and TUE and then categorised into the following groups, based on a classification derived from human medical studies [15,24,25] and our initial study in healthy dogs [35]:

VNC_HU_ − TUE_HU_ ≤ 5 HU (1)

VNC_HU_ − TUE_HU_ ≤ 10 HU (2)

VNC_HU_ − TUE_HU_ ≤ 15 HU (3)

VNC_HU_ − TUE_HU_ > 15 HU (4)

This categorisation relies on the human eye’s ability to detect a greyscale change of up to 6%, which equates to 24 shades of grey in an abdominal CT window (ranging from −150 HU to +250 HU) [43]. Even more conservative limits were adopted in human medicine studies for classifying HU differences between VNC and TUE images: differences of 10 HUs or less were considered negligible, whereas those between 10 and 15 HUs were deemed acceptable [15,25]. A difference of less than 5 HUs was included in the assessment to highlight the potential of the VNC technique.

### 2.5. Qualitative Image Analysis

The qualitative image analysis was performed by a board-certified veterinary radiologist (KM) and a veterinary radiologist in training (PL) in a consensus-based approach. A 5-point Likert scale was used to evaluate image quality. Using this scale, the level of image noise and overall quality was subjectively assessed by comparing spectral reconstruction images with conventional images, rated from 1 (markedly worse) to 5 (markedly better) (Table 3), and recorded for each patient.

A 5-point Likert scale was utilised to assess iodine subtraction from the VNC images compared to TUE (Table 4).

For each patient, the iodine subtraction in the liver (overall), liver (main lesion), spleen, pancreas, gallbladder, and muscle was evaluated, and an average score per patient and organ was calculated. The iodine subtraction was rated as insufficient, partly sufficient with larger areas of incomplete removal, moderately sufficient with incomplete areas in parts of the parenchyma, almost complete, and complete removal.

### 2.6. Histopathological Diagnosis

During the clinical workup of the included cases, histopathological samples were obtained through ultrasound-guided needle biopsy (Tru-Cut), biopsy during laparotomy, liver lobectomy, or necropsy. Soft tissue semi-automatic biopsy needles with adjustable penetration depth (BIO CORE 2, 16 G (1.67 mm × 10 cm), HVM Medical Products GmbH, Fulda, Germany) were used for the biopsies. The site for the Tru-Cut biopsies was chosen based on ultrasound imaging and was at the ultrasonographer’s discretion. The number of biopsies taken depended on the appearance of the lesions and the quality of the samples obtained (2–3 per patient). Due to the retrospective nature of the study, the location and number of Tru-Cut biopsies varied and were not standardised. The tissue samples were fixed in 10% buffered formalin, dehydrated in a graded ethanol series, and embedded in paraffin wax. Sections of 2–3 µm thickness were prepared and stained with haematoxylin-eosin. The histopathological examination was conducted by Diplomates of the European College of Veterinary Pathologists at the Institute for Pathology, University of Veterinary Medicine Hannover. The tissue samples were assessed based on the World Small Animal Veterinary Association Liver Standardisation Group criteria [44] and classified into seven groups, based on the predominant primary disease identified by the pathologists: degenerative changes, hepatitis, non-specific, adenoma, nodular hyperplasia, carcinoma, and blastoma of unspecified classification. In cases where the histopathological examination revealed features of two pathologies, patients were assigned to the predominant category; if inflammatory and degenerative features were present in equal measure, they were categorised as “non-specific”. If nodular hyperplasia was present along with hepatitis or degeneration, these patients were placed in the hepatitis or degeneration group, as nodular hyperplasia is typically an incidental finding without clinical relevance.

### 2.7. Statistical Analysis

Statistical analyses were performed using dedicated software (GraphPad Prism 10 for Windows, Version 10.2.3, GraphPad Software, San Diego, CA, USA; Microsoft^®^ Excel^®^ for Microsoft 365 MSO, Version 2406, Microsoft Corporation, Redmond, WA, USA). The equivalence of spectral-based VNC images and conventionally generated TUE scans was evaluated for each ROI by comparing the respective CT values (HU). To do this, differences were calculated by subtracting the HUs of each ROI in VNC from the matching ROI in TUE images. The resulting differences were categorised into four groups based on predefined cut-off values (difference ≤5 HUs, ≤10 HUs, ≤15 HUs, >15 HUs), with smaller differences indicating higher agreement between the two modalities. Additionally, two one-sided *t*-tests (TOST) were performed, using a null hypothesis that assumed the mean differences between VNC and TUE exceeded 5, 10, or 15 HUs, in order to assess the equivalence of the VNC and TUE series. To better focus on analysing different pathologies and lesion types, all measured ROIs in each category were grouped together and analysed independently of the number of subjects. A *p*-value less than 0.05 was considered statistically significant, leading to the rejection of the null hypothesis and indicating that the attenuation of VNC and TUE at this threshold was equivalent.

To evaluate measurement consistency, coefficients of variation (CV) were calculated. For intra-lesion variability, the CV of HU within each ROI was determined for each lesion. Results were presented by group (multifocal, focal, diffuse) and separately for VNC and TUE as median and interquartile range (IQR, 25th–75th percentile). For inter-lesional variability, a single CV was computed per group (multifocal, focal, diffuse) based on the mean attenuation values of all lesions within each group, reflecting the variability between lesions.

## 3. Results

### 3.1. Study Population

During the study period from July 2021 to July 2023, a total of 433 abdominal SDCT examinations of dogs were conducted at the Department of Small Animal Medicine and Surgery at the University of Veterinary Medicine Hannover. Of these, 31 dogs met the inclusion criteria and were initially enrolled in the study. However, three cases were excluded due to incomplete spectral data collection, leaving 28 dogs meeting the inclusion criteria. The breeds included were: mixed breed dogs (8), Golden Retriever (4), Labrador Retriever (2), Dachshund (2), Rhodesian Ridgeback (2), Husky (2), Australian Shepherd (1), Australian Cattle Dog (1), Irish Setter (1), Rottweiler (1), Galgo Espanol (1), Standard Poodle (1), Fox Terrier (1), and Coton de Tulear (1). The average age of the dogs was 10 years, ranging from 7 months to 14 years and 4 months; there were 14 female dogs (9 spayed) and 14 male dogs (7 neutered).

### 3.2. Histopathological Findings

In 14 cases, liver samples were obtained through ultrasound-guided Tru-Cut biopsies; 10 biopsies were carried out surgically during laparotomy, three dogs underwent liver lobe resections, and a necropsy was performed in one case. Based on the results of the histopathological examination of the samples, the patients were classified into the seven groups described above according to the predominant primary disease.

Twenty-two out of 28 patients showed benign liver changes. A histopathological diagnosis revealed predominantly degenerative alterations in nine dogs. Hydropic degeneration, characterised by swollen, vacuolated hepatocytes, was seen in eight of these nine cases. Additionally, six livers had a buildup of various pigments, such as bile pigments, lipofuscin, or haemosiderin. Only one liver displayed degenerative changes linked to a hepatic metabolic storage disorder without hydropic degeneration. The second most frequent change was hepatitis, found in seven dogs. In three cases, it was difficult to differentiate between degenerative and inflammatory changes because features of both were present; thus, these were classified as non-specific. A hepatic adenoma was identified in two dogs during the histopathological examination, while one dog showed nodular hyperplasia without further inflammatory or degenerative changes.

Of the six malignant neoplasms, four were carcinomas and two were blastomas of unspecified type. All four carcinomas were primary tumours, comprising three hepatocellular carcinomas and one bile duct carcinoma.

### 3.3. Imaging Features of Liver Lesions

The evaluation of CT studies in the 28 dogs revealed 18 with multifocal, 6 with focal, and 4 with diffuse hepatopathies. Of the 18 characterised as multifocal, histopathology showed degenerative changes (8), hepatitis (3), non-specific (2), adenoma (2), nodular hyperplasia (1), blastoma (1), and carcinoma (1). The six dogs with a single focal liver lesion had carcinomas (3), hepatitis (2), and blastoma (1). The four livers with diffuse alterations were classified as hepatitis (2), degenerative (1), and non-specific (1).

In 13 cases, all lobes of the liver were affected. Five dogs exhibited alterations in a single hepatic lobe. Compared to the surrounding tissue in pre-contrast images, 16 lesions were hypoattenuating, three were isoattenuating, one was hyperattenuating, and four displayed mixed attenuation within the same lesion or across several lesions. None of the characterised lesions showed mineralisation. One hepatic lesion had a capsule, which was diagnosed as hepatocellular carcinoma upon histopathological examination. Of the examined organs, 20 showed no cavitation, six had one cavitary lesion (carcinomas (3), adenoma (1), degeneration (1), hepatitis (1)), and two had multiple cavitary lesions (carcinoma (1), adenoma (1)). In the post-contrast images, the attenuation of the focal or multifocal lesions compared to surrounding tissue was hypoattenuating in 14 cases, hyperattenuating in one, and exhibited mixed attenuation in 9 cases, either within the same lesion or across multiple lesions. The contrast enhancement pattern of the liver lesions was described as homogeneous in 11 cases, heterogeneous in 12, and in five cases, different enhancement patterns were observed across various lesions. No circular peripheral enhancement patterns, known as target lesions, were evident.

### 3.4. Quantitative Image Analysis

**Figure 4:**
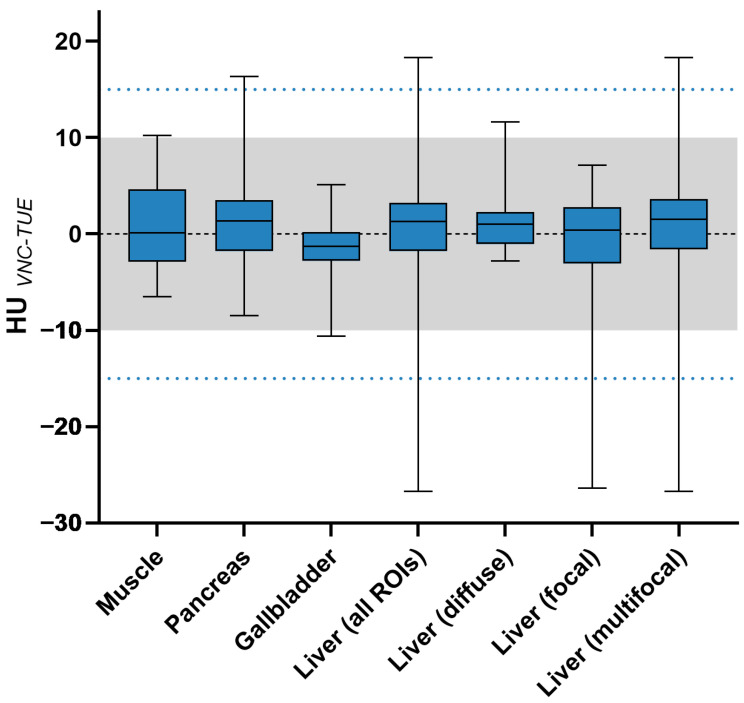
Difference between VNC and TUE in HUs for all liver ROIs, categorised according to imaging features, as well as the indicator organs. The threshold of 10 HUs difference (“negligible”) is indicated by the grey shaded area, while the threshold of 15 HUs difference (“acceptable”) is represented by the blue dotted lines. The lower and upper margins of each box indicate the 25th and 75th percentiles, respectively. The median is marked by a black line within the plots.

**Figure 5:**
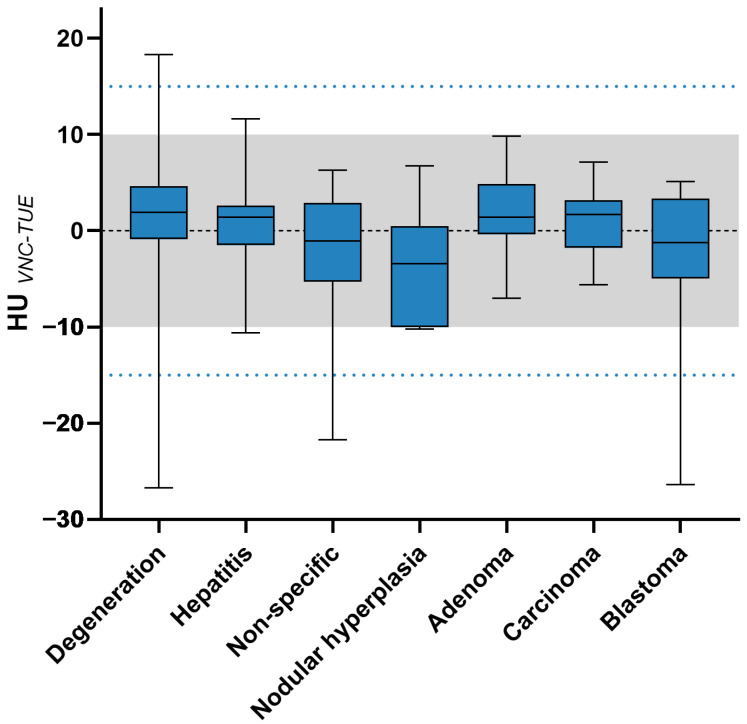
Difference between VNC and TUE in HUs for all ROIs of the liver, categorised by histopathological diagnosis. The threshold of 10 HUs difference (“negligible”) is shown by the grey shaded area, while the threshold of 15 HUs difference (“acceptable”) is illustrated by the blue dotted lines. The lower and upper margins of each box represent the 25th and 75th percentiles. A black line within the plots indicates the median.

To evaluate the equivalence of spectral-based VNC and TUE images, 287 ROIs were analysed quantitatively, resulting in 574 Hounsfield unit values. The differences between TUE and VNC for the indicator organs, muscle, pancreas, and gallbladder—as well as for the liver in general—are shown in Table 5, along with Figure 4 and Figure 5, differentiated according to imaging features and histopathological diagnosis.

The difference in HUs between VNC and TUE images was ≤15 in all patients for both the muscle and the gallbladder. One ROI measured in the pancreas of a patient with hepatitis was slightly above the upper limit of 15 HUs difference, recording a value of 16.3 HUs, resulting in an overall percentage for the pancreas of 96.43% having ≤15 HUs difference. In the additionally measured organs (paraspinal muscle, pancreas, and gallbladder content), the difference between HUs in VNC and TUE images was 96.43% ≤10. A threshold of ≤5 HUs difference was applied to 71.43% for muscle, 85.71% for pancreas, and 92.86% for gallbladder.

The differences were ≤5 in 75.86%, ≤10 in 92.61%, and ≤15 in 97.54% for all calculated ROIs in the liver. The differences in HUs were examined in groups based on the imaging type of liver lesions and the histopathological diagnoses. According to the classification based on the lesion’s imaging type, the group with diffuse lesions reached 100%, focal lesions 97.5%, and multifocal lesions 97.12% with a ≤15 HUs difference. VNC performed well in pre-contrast hyper- or hypoattenuating lesions with 100% accuracy for ≤10 HUs difference, as well as in cavitary lesions with similar results. VNC showed comparable performance at the periphery of malignant lesions compared to other metrics in malignant lesions, with a ≤10 HUs difference noted in all six patients with malignant neoplasms. When patients were categorised by the primary disease determined by pathologists, groups with diagnoses of hepatitis, adenoma, nodular hyperplasia, and carcinoma all achieved 100%, while degenerative changes reached 95.89%, non-specific changes 94.12%, and blastoma 92.86% for a ≤15 HUs difference. Of the 287 ROIs quantitatively assessed in the study, six ROIs exhibited a difference greater than 15 HUs between the determined TUE and VNC values. One of these ROIs, located in the pancreas of a patient in the hepatitis group, had a difference of 16.3 HUs, just above the threshold. Furthermore, three patients presented with degenerative changes and one with non-specific (but benign) changes, all showing a multifocal distribution pattern of their lesions without mineralisation or cavitations. The ROIs displaying differences exceeding the 15 HUs threshold were all small lesions in three patients, which were entirely encompassed in one ROI during the measurement of multiple small lesions. Another patient, classified as degenerative in histopathology, had one ROI with a difference greater than 15 HUs between VNC and TUE images in the normally appearing hepatic parenchyma. A dog diagnosed with blastoma also had an additional small cystic lesion that did not perform well in VNC, showing a difference of 26 HUs from the TUE value.

The two one-sided *t*-tests (TOST) confirmed the equivalence of TUE and VNC for all ROIs and, consequently, patients, within the limit of ≤10 HUs, with a *p*-value of <0.05 (Table 6).

**Figure 6:**
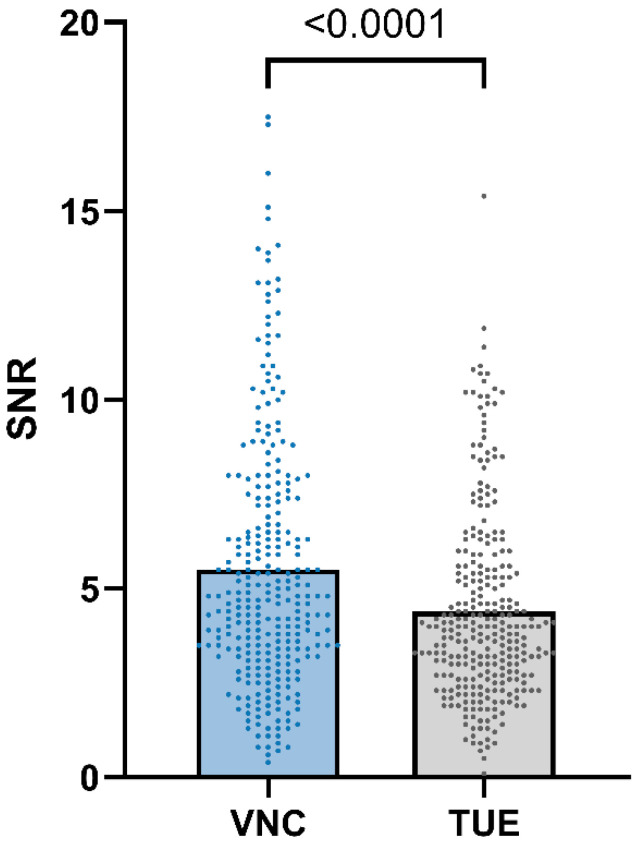
Signal-to-noise ratio (SNR) of VNC images compared to TUE images. The SNR was calculated by dividing the HUs by the associated standard deviation, indicating that the signal intensity and, therefore, the image quality increase proportionally to the SNR value. The *p*-value of <0.0001 confirmed that the SNR was significantly better for VNC image data than for TUE image data.

SNR (VNC) was equal to or higher than SNR (TUE) in 84.66% of the ROIs, as shown in Figure 6.

The intra-lesional CVs within the ROIs were relatively similar between VNC and TUE images. Median (IQR) intra-lesional CVs were 16.25% (6.99–26.97%) for multifocal lesions in VNC and 17.74% (7.15–26.88%) in TUE; 13.99% (5.94–18.78%) for focal lesions in VNC and 13.07% (2.26–20.96%) in TUE; and 6.62% (3.03–10.2%) for diffuse hepatic changes in VNC and 7.33% (6.04–8.66%) in TUE. The inter-lesional CVs were 30.51% (multifocal), 25.4% (focal), and 15.02% (diffuse) in VNC images, and 28.1%, 15.58%, and 13.9% in TUE images.

### 3.5. Qualitative Image Analysis

The 5-point Likert scale for image quality resulted in an average score of 3.96 (SD ± 0.33). Two patients received a score of 3, indicating comparable image quality, while all other patients scored either 4 (25) or 5 (1), with reduced noise and improved homogeneity of the displayed tissues. The 5-point Likert scale for iodine subtraction showed an average of 3.86 (SD ± 0.65) for the liver, with complete removal of contrast medium (score 5) in 4 cases and nearly complete removal (score 4) in 16 cases. Eight patients demonstrated moderate removal of contrast medium, with incomplete areas in parts of the parenchyma (score 3). For the hepatic main lesion, the average score of 4.25 (SD ± 0.68) was derived from the iodine subtraction scale; most patients were rated with scores of 4 (8) or 5 (6), with only two patients scoring 3. The average scores for the indicator organs were as follows: spleen 4 (SD ± 0.94), pancreas 4.79 (SD ± 0.42), gallbladder 4.82 (SD ± 0.39), and muscle 5.0 (SD ± 0). The iodine subtraction scores and the image quality scores for the included dogs are presented in Table 7.

## 4. Discussion

Our study aimed to assess the equivalence of Hounsfield units calculated from spectral-based VNC images compared to TUE images in hepatic pathologies in dogs. In 92.61% of all hepatic ROIs analysed in our study, differences in attenuation values between VNC and TUE were ≤10 HUs, which is considered negligible in the literature [15].

Considering a limit of ≤15 HUs difference as acceptable, the percentage of ROIs meeting this criterion could be increased to 97.54%. These results were similar to those obtained by Sauter et al. [15] and Jamali et al. [24] in their studies on humans and aligned with the findings of Lietz et al. in their study on the performance of VNC in healthy dogs [35]. The muscle, pancreas, and gallbladder were included, as higher differences in HUs between VNC and TUE in these organs compared to our progenitor study would suggest possible technical errors. Although we had to consider the potential for pathological changes in these organs, no abnormalities were detected during the evaluation of the CT scans. Muscle, pancreas, and gallbladder content achieved a value of 96.43% for ≤10 HUs, which is also comparable to the results from the previously mentioned studies, where an identical type of CT scanner was used. It seems reasonable that the performance of VNC depends on distribution pattern, accumulation, concentration of iodine in the tissue, and interindividual variability, as previously discussed in human medicine [45].

Based on the outcomes of our study, we confirmed that the VNC calculations showed excellent accuracy in liver pathologies, as well as muscle, pancreas, and gallbladder content. Using VNC images in a clinical setting can potentially halve the radiation dose, shorten scan times, and reduce the duration of general anaesthesia. This is especially advantageous for unstable patients and, more broadly, for patients with an increased risk related to anaesthesia.

When reviewing the differences in HUs based on their imaging features, it was evident that the results were relatively similar, with a difference of ≤15 HUs between VNC and TUE values in 100% of cases for diffuse, 97.5% for focal, and 97.12% for multifocal pathologies. However, when using a limit of ≤10 HUs, the focal pathologies performed better than the other two categories. Despite the varying patient distribution, one possible reason for the better performance of focal lesions is that ROIs could be placed more accurately compared to the multifocal group, which included some patients with rather small lesions. In the case of focal lesions, one polygonal ROI covering the entire lesion was drawn, along with two ROIs at the periphery and two at the centre of the lesion. Notably, four of the six ROIs with VNC differences >15 HUs belonged to smaller lesions, each represented by a single ROI. One such small lesion, previously suspected to be a cyst, was measured alongside a blastoma in patient number 20. This lesion appeared hypoattenuating compared to the surrounding hepatic tissue in both TUE and post-contrast images and showed no iodine uptake. An error in the reconstruction algorithm was previously suspected by Sauter et al. for a similar lesion [15]. While cystic lesions are easily identifiable in CT scans, caution should be exercised regarding potential errors in the VNC algorithm concerning these lesions.

Since we were also interested in whether cavitary lesions pose a problem for the reconstruction algorithm, these were reviewed as a group, comprising 40 ROIs, all of which were below the threshold of a 10 HUs difference. Consequently, VNC was not affected by the cavitary nature of a lesion. We also aimed to investigate whether issues arose in VNC images due to the phenomenon of neovascularisation in the periphery of malignant tumours, as previous studies [22,24] have described inadequate iodine subtraction from small intraparenchymal vessels by the reconstruction algorithm. Laukamp et al. [22] discussed the combination of small vessel size and high contrast media concentrations as the cause of this problem, since their study indicated that iodine subtraction was sufficient in the aorta, a larger vessel. To assess this in the six patients diagnosed with malignant tumours, the 12 ROIs located at the margins of the lesions were examined individually, with 100% showing a difference of ≤10 HUs between VNC and TUE. The small number of malignant tumours in our study means that a definitive conclusion is impossible; nonetheless, there is no evidence of reduced iodine subtraction success in this region.

If patients were grouped and evaluated based on histopathological diagnosis, the categories of hepatitis, nodular hyperplasia, carcinoma, and adenoma showed the best performance, with a difference of 100% ≤15 HUs. Lesions classified as degenerative, non-specific, or blastoma also ranged between 92.86% and 95.89%, as shown in Table 5. With a lower threshold value of ≤10 HUs difference, the carcinoma and adenoma groups distinguished themselves, continuing to reach 100%. Furthermore, the blastoma group could be included, as it achieved 100% for a ≤10 HUs difference, provided the previously described cystic lesion in patient number 20 was excluded. These results are promising for using the VNC technique in clinical patients.

Various studies have shown that the VNC algorithm performed poorly for fat, as VNC tended to overestimate HUs compared to unenhanced images [15,24,25,35,46]. A potential explanation is that fat is not included in the material decomposition algorithm, which involves the iodine and water pair used in the VNC reconstruction [47]. We were unable to replicate these previous findings in this study because none of the patients had a histopathological diagnosis of hepatic steatosis, and subcutaneous fat was not measured. The suitability of the VNC technique for these conditions still needs to be assessed in future research and may pose a potential challenge.

Another challenge of VNC images that we could not investigate in our study was mineralisation, as no mineralised lesions were found in our patients. Mangold et al. [48] previously pointed out the risk that mineralisation might go unnoticed due to excessive or insufficient subtraction of residual iodine, especially for structures smaller than 3 mm. A recently published study from our research group also exhibited a potential drawback of the VNC algorithm in mineralised lesions in splenic pathologies [36]. This will be particularly relevant when exploring the use of the VNC technique for the interpretation of malignant neoplasia and renal and lower urinary tract pathologies. If we were able to obtain more information on the potential difficulties of the VNC algorithm with fat-attenuation lesions and mineralisations, the native scan might be omitted in the near future.

We only evaluated images acquired in the portal venous phase, as these were available for all patients and the liver parenchyma shows a more homogeneous enhancement in this phase, enabling probably more accurate VNC reconstruction. It is controversially discussed in the literature whether there are differences between the performance of VNC in the arterial and venous phase [15,25,49,50]. VNC reconstruction in humans is commonly performed from venous-phase datasets, yielding HU values that closely approximate true non-contrast images while preserving image quality [23,24,51,52].

In the second part of our study, we evaluated image quality based on image noise and iodine subtraction in SDCT images through qualitative analysis. The subjective assessment showed that the image quality of SDCT images was better than that of conventional CT images in most dogs (26 out of 28) and equivalent in the remaining two cases. Additionally, SDCT performed better in terms of SNR for most subjects. Therefore, our hypothesis regarding the superior image quality of SDCT was confirmed by our findings. The objectively and subjectively improved image quality of SDCT images is likely caused by the reduced noise, improved temporal and spatial resolution and artefact reduction, which has been proven in previous studies in human [53] and veterinary medicine [35,36,37]. The iodine subtraction also performed well in the subjective evaluation, achieving an average score of 3.86 for the liver and scores of at least 4 for reference organs such as the spleen, pancreas, gallbladder, and muscle. Compared to the study by Lietz et al. [35], the liver and spleen received slightly lower scores when using the same evaluation criteria, while the scores for the pancreas remained similar. The authors suggested that hepatic diseases and, in some cases, additional splenic pathologies may have contributed to these marginally lower scores compared to the study involving healthy dogs. We aimed to perform both objective and subjective quality assessments to validate our clinical impression of an overall improvement in SDCT image quality.

The relatively high intra- and inter-lesional CVs, particularly in multifocal and focal lesions, reflect the inherent heterogeneity of hepatic pathologies. Variability was lower in diffuse hepatic changes, probably due to more uniform parenchymal involvement. The relative similarity of the CVs between VNC and TUE images, regardless of their absolute values, emphasises the comparability of both techniques. 

The present study has several limitations. Firstly, the patient population was relatively small, consisting of 28 dogs, which limited our ability to draw conclusions regarding the influence of breed, sex, age, and body condition. Breed and sex influence body size and thus the field of view; age may affect metabolic conditions, and body condition could influence parenchymal fat, the potential pitfall of the VNC algorithm. The distribution of hepatic pathologies was uneven, with only six malignant tumours and just one case of nodular hyperplasia as the predominant disease. Due to the small group sizes, the informative value of our results is limited. In some cases, histopathology revealed features of both degenerative and inflammatory changes. Patients were classified into this category if one change was clearly dominant; if both were present equally, they were categorised as “non-specific.” Since all pathologies scored higher than 90% for a difference of ≤15 HUs, the type or combination of pathologies likely did not significantly affect the performance of the VNC algorithm. Because of the varied presentations of the pathologies, ROI placement, despite our established criteria, was challenging. Particularly, ROI placement in smaller and multifocal lesions proved more difficult compared to larger focal lesions and probably introduced variability. Unfortunately, our study did not include patients with steatitis or parenchymal mineralisations. These pathologies could be of particular interest, as previously discussed, and should be considered in future research. Lastly, as the technical specifications may vary between vendors, the type of scanner might influence the results. Greffier et al. discuss the currently available spectral CT scanner [54].

## 5. Conclusions

In conclusion, we observed high equivalence in attenuation measurements between VNC and TUE images in dogs with hepatic pathologies, with a negligible difference of ≤10 HUs in 92.61% of all ROIs placed in the hepatic parenchyma. Among the various groups, VNC performed best for focal lesions. The image quality of SDCT-derived images was comparable to or better than that of conventional CT images in qualitative assessments, and the SNR was significantly higher for VNC images. Therefore, VNC images generated from SDCT data could potentially serve as an alternative to conventional TUE images for hepatic pathologies. However, larger studies with more balanced distributions of pathologies, along with further research on the hepatic conditions of different abdominal organs, are necessary before the TUE scan can be omitted in clinical patients.

## Figures and Tables

**Table 1:** Evaluation criteria in pre- and post-contrast conventional CT.

Evaluation Criteria	Classifications
Peritoneal fluid	None, mild, moderate, severe
Surface of the liver	Smooth, irregular
Contours of the liver (apart from the lesion)	Sharp angulation, rounded borders
Lesion type	Diffuse, focal, multifocal
Number of lesions	1, 2–5, 5–10, >10
Localisation of lesions	Lobus hepatis sinister lateralis/medialis, Lobus quadratus, Lobus hepatis dexter lateralis/medialis, Lobus caudatus, all lobes
Shape of lesions	Round, oval, amorphous, and different types of shapes of multifocal lesions
Border of lesions	Irregular, regular
Margination of lesions	Well-defined, ill-defined, different for different lesions
Extent of lesions	Intraparenchymal, extending over the hepatic border, both types of lesions present
Size of lesions	Maximum extension in cm (applies to the largest lesion, measured in all three planes)
Capsule formation	No, yes
Cavitation	No, one lesion, several lesions
Attenuation pre-/post-contrast compared to the surrounding parenchyma	Hypoattenuating, isoattenuating, hyperattenuating, and different attenuations within the same lesion or several lesions
Enhancement pattern	Homogeneous, heterogeneous, mainly peripheral, circular peripheral (target lesions), different types of enhancement in different lesions
Degree of enhancement	Mild, moderate, severe, different within the same lesion or in different lesions
Mineralisations	None, mild, moderate, severe
Size of portal lymph nodes	Normal, mild/moderate/severe enlargement
Structure of portal lymph nodes	Homogeneous, heterogeneous
Enhancement pattern of portal lymph nodes	Homogeneous, heterogeneous

**Table 2:** Placement of ROIs on the hepatic tissue depending on CT characterisation.

Lesion Type Based on Imaging Characteristics	ROI Placement
Diffuse	Left lateral lobe (1 ROI) Left medial lobe (1 ROI) Right lateral lobe (1 ROI) Right medial lobe (1 ROI) Central (2 ROIs)
Focal	Entire lesion (1 polygonal ROI) Periphery of the lesion (2 ROIs) Centre of the lesion (2 ROIs) Normal Parenchyma (2 ROIs)
Multifocal (main lesion and additional lesions)	Main lesion: -Entire lesion (1 polygonal ROI) -Periphery of the lesion (2 ROIs) -Centre of the lesion (2 ROIs) Additional (mineralised) lesion: -Entire lesion (1 polygonal ROI) -Periphery of the lesion (2 ROIs) -Centre of the lesion (2 ROIs) Normal appearing parenchyma (2 ROIs)
Multifocal (only multiple small lesions)	1 ROI per lesion (4 ROIs) Normal appearing parenchyma (2 ROIs)

**Table 3:** 5-point Likert-scale for assessment of image noise and image quality in spectral-based image (SBI) reconstructions compared to conventional images.

Image Noise and Image Quality: SBI Reconstructions vs. Conventional CT Images	
1	SBI reconstructions markedly worse than conventional CT images
2	SBI reconstructions mildly worse than conventional CT images
3	SBI reconstructions equivalent to conventional CT images
4	SBI reconstructions mildly better than conventional CT images
5	SBI reconstructions markedly better than conventional CT images

**Table 4:** 5-point Likert scale for assessment of iodine subtraction from VNC images compared with TUE.

Parenchymal Iodine Subtraction in VNC	
1	Insufficient subtraction of contrast medium
2	Partly sufficient removal of contrast medium with larger, incomplete areas
3	Moderate removal of contrast medium with incomplete areas in parts of the parenchyma
4	Almost complete removal of contrast medium
5	Complete removal of contrast medium

**Table 5:** Number (percentage) of ROIs, divided by their localisation, imaging characteristics and histopathological diagnosis, with a difference in TUE and VNC attenuation values ≤15, ≤10, or ≤5.

Region of Interest (All ROIs)	≤5		≤10		≤15	
Localisation						
Muscle	20/28	71.43%	27/28	96.43%	28/28	100%
Pancreas	24/28	85.71%	27/28	96.43%	27/28	96.43%
Gallbladder	26/28	92.86%	27/28	96.43%	28/28	100%
Liver	154/203	75.86%	188/203	92.61%	198/203	97.54%
Imaging characteristics						
Diffuse	21/24	87.5%	22/24	91.67%	24/24	100%
Focal	35/40	87.5%	39/40	97.5%	39/40	97.5%
Multifocal	98/139	70.5%	127/139	91.37%	135/139	97.12%
Cavitary lesions	34/40	85%	40/40	100%	40/40	100%
Periphery of malignant neoplasia	8/12	66.67%	12/12	100%	12/12	100%
Histopathological diagnosis						
Degeneration	51/73	69.86%	65/73	89.04%	70/73	95.89%
Hepatitis	42/49	85.71%	46/49	93.88%	49/49	100%
Non-specific	11/17	64.7%	15/17	88.24%	16/17	94.12%
Nodular hyperplasia	3/6	50%	5/6	83.33%	6/6	100%
Adenoma	12/16	75%	16/16	100%	16/16	100%
Carcinoma	25/28	89.29%	28/28	100%	28/28	100%
Blastoma	10/14	71.43%	13/14	92.86%	13/14	92.86%

**Table 6:** Test for equivalence using two one-sided *t*-tests (TOST).

Categories (All ROIs)	≤5 HUs	≤10 HUs	≤15 HUs
Muscle (*n* = 28)	0.0035	<0.0001	<0.0001
Pancreas (*n* = 28)	0.0077	<0.0001	<0.0001
Gall bladder (*n* = 28)	<0.0001	<0.0001	<0.0001
Liver (*n* = 203)	0.0002	<0.0001	<0.0001
—diffuse (*n* = 37)	0.0533	<0.0001	<0.0001
—one lesion (*n* = 40)	0.0118	<0.0001	<0.0001
—multifocal (*n* = 126)	0.0122	<0.0001	<0.0001
—degenerative (*n* = 73)	0.232	<0.0001	<0.0001
—hepatitis (*n* = 49)	<0.0001	<0.0001	<0.0001
—non-specific (*n* = 17)	0.4852	0.0005	<0.0001
—hyperplasia (*n* = 6)	0.7105	0.0213	0.0009
—adenoma (*n* = 16)	0.0121	<0.0001	<0.0001
—carcinoma (*n* = 28)	<0.0001	<0.0001	<0.0001
—blastoma (*n* = 14)	0.5845	0.0098	<0.0001
All ROIs (*n* = 287)	<0.0001	<0.0001	<0.0001

**Table 7:** Iodine Subtraction score and Image Quality/Image Noise score for all dogs included in the study.

	Patient Nr.														
	1	2	3	4	5	6	7	8	9	10	11	12	13	14	
**Location**	**Score: Iodine Subtraction**														**Average Score (Location)**
Liver (overall)	3	3	5	3	5	4	4	3	4	3	3	4	4	4	3.71
Liver (main lesion)				4			5	4	5	4		3	5		4.29
Spleen	3	3	5	3	5	4	5	4	3	2	4	5		3	3.77
Pancreas	4	4	5	5	5	5	5	5	4	5	5	5	5	4	4.71
Gallbladder	5	5	5	4	5	5	5	5	5	5	4	5	5	5	4.86
Muscle	5	5	5	5	5	5	5	5	5	5	5	5	5	5	5
**Average Score (Patient)**	4	4	5	4	5	4.6	4.83	4.33	4.33	4	4.2	4.5	4.8	4.2	4.4
	**Score: Image Quality/Image Noise**														**Average Score (Quality)**
	4	4	4	4	3	4	4	4	4	4	5	4	4	4	4
	**Patient Nr.**														
	15	16	17	18	19	20	21	22	23	24	25	26	27	28	
**Location**	**Score: Iodine Subtraction**														**Average Score (Location)**
Liver (overall)	4	4	4	3	5	4	4	4	4	3	4	4	5	4	4
Liver (main lesion)		4	4		5		3		4	4		4	5	5	4.22
Spleen	4	5	5	3	5	5	3	3	4	5	4	5	4		4.23
Pancreas	5	5	5	4	5	5	5	4	5	5	5	5	5	5	4.86
Gallbladder	4	5	5	5	4	5	5	5	5	4	5	5	5	5	4.79
Muscle	5	5	5	5	5	5	5	5	5	5	5	5	5	5	5
**Average Score (Patient)**	4.4	4.67	4.67	4	4.83	4.8	4.17	4.2	4.5	4.33	4.6	4.67	4.83	4.8	4.52
	**Score: Image Quality/Image Noise**														**Average Score (Quality)**
	4	4	3	4	4	4	4	4	4	4	4	4	4	4	3.93

## Data Availability

Data are available on reasonable request from the authors.

## Abbreviations

The following abbreviations are used in this manuscript:

CT	computed tomography
DECT	dual-energy computed tomography
HU	Hounsfield Unit
keV	kilo-electronvolts
KM	Kristina Merhof
LC	Lydia Claußen
PL	Philipp Lietz
ROI	region of interest
SBI	spectral-based images
SD	standard deviation
SDCT	spectral detector computed tomography
SNR	signal-to-noise ratio
TOST	two one-sided t-tests
